# Effectiveness and Safety of Antithrombotic Medication in Patients With Atrial Fibrillation and Intracranial Hemorrhage: Systematic Review and Meta-Analysis

**DOI:** 10.1161/STROKEAHA.122.038752

**Published:** 2022-07-08

**Authors:** Elena Ivany, Leona A. Ritchie, Gregory Y.H. Lip, Robyn R. Lotto, David J. Werring, Deirdre A. Lane

**Affiliations:** Liverpool Centre for Cardiovascular Science (E.I., L.A.R., G.Y.H.L., R.R.L., D.A.L.); Department of Cardiovascular and Metabolic Medicine, Institute of Life Course and Medical Sciences (E.I., L.A.R., G.Y.H.L., D.A.L.), University of Liverpool, United Kingdom.; Liverpool Heart and Chest Hospital, United Kingdom (G.Y.H.L., D.A.L.).; Department of Clinical Medicine, Aalborg University, Denmark (G.Y.H.L., D.A.L.).; School of Nursing and Allied Health, Faculty of Health, Liverpool John Moores University, United Kingdom (R.R.L.).; Stroke Research Centre, University College London, Queen Square Institute of Neurology, United Kingdom (D.J.W.).

**Keywords:** anticoagulant, atrial fibrillation, intracranial hemorrhage, ischemic stroke, systematic review

## Abstract

For patients with atrial fibrillation who survive an intracranial hemorrhage (ICrH), the decision to offer oral anticoagulation (OAC) is challenging and necessitates balancing risk of thromboembolic events with risk of recurrent ICrH.

**Methods:**

This systematic review assesses the effectiveness and safety of OAC and/or antiplatelets in patients with atrial fibrillation with nontraumatic ICrH. Bibliographic databases CENTRAL, MEDLINE, EMBASE, and CINAHL were searched. Articles on adults with atrial fibrillation with spontaneous ICrH (intracerebral, subdural, and subarachnoid), receiving antithrombotic therapy for stroke prevention were eligible for inclusion.

**Results:**

Twenty articles (50 470 participants) included 2 randomized controlled trials (n=304)‚ 8 observational studies, 8 cohort studies, and 2 studies that meta-analyzed individual-level data from observational studies. OAC therapy was associated with a significant reduction in thromboembolic events (summary relative risk [sRR], 0.51 [95% CI, 0.30–0.86], heterogeneity I^2^=2%; *P*=0.39, n=5 studies) and all-cause mortality (sRR, 0.52 [95% CI, 0.38–0.71], heterogeneity I^2^=0; *P*=0.44, n=3 studies). OAC therapy was not associated with an increased risk of recurrent ICrH (sRR, 1.44 [95% CI, 0.38–5.46], heterogeneity I^2^=70%, *P*=0.02, n=5 studies). Nonvitamin K antagonist OACs were more effective at reducing the risk of thromboembolic events (sRR, 0.65 [95% CI, 0.44–0.97], heterogeneity I^2^=72%, *P*=0.03, n=3 studies) and were associated with a lower risk of recurrent ICrH (sRR, 0.52 [95% CI, 0.40–0.67], heterogeneity I^2^=0%, *P*=0.43, n=3 studies) than warfarin.

**Conclusions:**

In nontraumatic ICrH survivors with atrial fibrillation, OAC therapy is associated with a reduced risk of thromboembolic events and all-cause mortality without significantly increasing risk of recurrent ICrH. This finding is primarily based on observational data, and further larger randomized controlled trials are needed to corroborate or refute these findings.

Long-term oral anticoagulation (OAC) is the main treatment for ischemic stroke prevention in patients with atrial fibrillation (AF) and at least 1 additional stroke risk factor,^1^ but all OAC therapy is associated with an increased risk of bleeding. Intracranial hemorrhage (ICrH) is a potential complication of OAC^2^ and is associated with significant mortality and morbidity.^3,4^ ICrH survivors are at risk of sustaining further hemorrhage or an ischemic stroke, particularly if AF is present.^5,6^ As a result, the clinical dilemma about what, if any, stroke prevention therapy should be offered to ICrH survivors with AF persists. There are few published data from randomized controlled trials (RCTs),^7,8^ and most of the available evidence is from observational studies.

This review aims to systematically assess the effectiveness and safety of OAC in patients with AF who have sustained a nontraumatic ICrH.

## Methods

### Data Availability

The data that support the findings of this study are available from the corresponding author upon reasonable request.

### Eligibility Criteria

The review was conducted in accordance with the Preferred Reporting Items for Systematic reviews and Meta-Analyses (PRISMA) guidelines and was registered with the PROSPERO database of systematic reviews (https://www.clinicaltrials.gov; Unique identifier: CRD42020223266).

### Participants

To be eligible for inclusion, articles had to report on adults (aged ≥18 years) with AF who had survived a nontraumatic spontaneous ICrH of any size and any type (lobar, brain stem, deep, cerebellar, subdural, epidural or subarachnoid location; see Table S1 for definitions of terms) or had cerebral microbleeds.

### Intervention

The intervention of interest was long-term OAC and/or antiplatelets for stroke prevention in AF. Short-term and/or nonoral anticoagulation therapy or OAC for other reasons were excluded.

### Comparators

Any form of oral, long-term anticoagulation therapy and/or antiplatelet therapy, or no comparator (no therapy) were considered.

### Outcomes

The primary outcomes were thromboembolic events and recurrent ICrH. Thromboembolic events were chosen as an outcome to reflect the range of definitions used in the included studies (eg, ischemic stroke and/or systemic embolism, and thromboembolic events). Secondary outcomes were major bleeding, all-cause mortality, cardiovascular mortality, fatal hemorrhage or stroke, incidence of clinically significant nonmajor bleeding or thromboembolic events (other than ischemic stroke).

### Search Strategy

The following electronic databases were searched: CENTRAL (29/06/20, 07/12/20, and 25/10/21), MEDLINE (03/07/20, 25/10/20, and 25/10/21), EMBASE and CINAHL (30/06/20, 25/09/20, and 25/10/21). Search terms and index terms associated with AF, intracerebral hemorrhage (ICH), major bleeding, and anticoagulant medications were included (Table S2). Only full-text articles were included. Searches were not limited by language but were restricted to the year 2000 onward.

### Study Selection

Two researchers (E.I. and L.A.R.) independently assessed the suitability of articles for inclusion against the eligibility criteria. Any disagreements were resolved through examination of the original data and discussion, with recourse to a third reviewer (D.A.L.) where necessary.

### Data Extraction

Two researchers (E.I. and L.A.R.) extracted relevant data from the articles using a standardized tabulated data extraction form. One author provided additional unpublished data.^9^

### Risk of Bias Assessment

Observational studies were assessed using the Risk of Bias Assessment Tool for Nonrandomized Studies (RoBANS)^10^ (Figure S1) and RCTs were assessed using the Cochrane Collaboration’s tool for assessing risk of bias^11^ (Figure S2) independently by 2 researchers (E.I. and L.A.R.).

### Data Synthesis

Included studies were assessed for clinical and statistical heterogeneity. Meta-analyses were performed if studies reported similar designs, had the same outcomes, comparable interventions and comparators, and pooling the results was appropriate. Studies that could not be included in meta-analyses are reported narratively.

### Statistical Analysis

A random effects model was used in all meta-analyses. Event data for control and intervention groups was compared using risk ratios and associated 95% CIs. Sensitivity analyses were performed according to outcome and follow-up period, where appropriate.

Statistical heterogeneity was evaluated using the I^2^ statistic. An I^2^ value of 0% to 40% indicated low heterogeneity, 30% to 60% moderate heterogeneity, 50% to 90% substantial heterogeneity, and ≥75% considerable heterogeneity.

## Results

**Figure 1.**
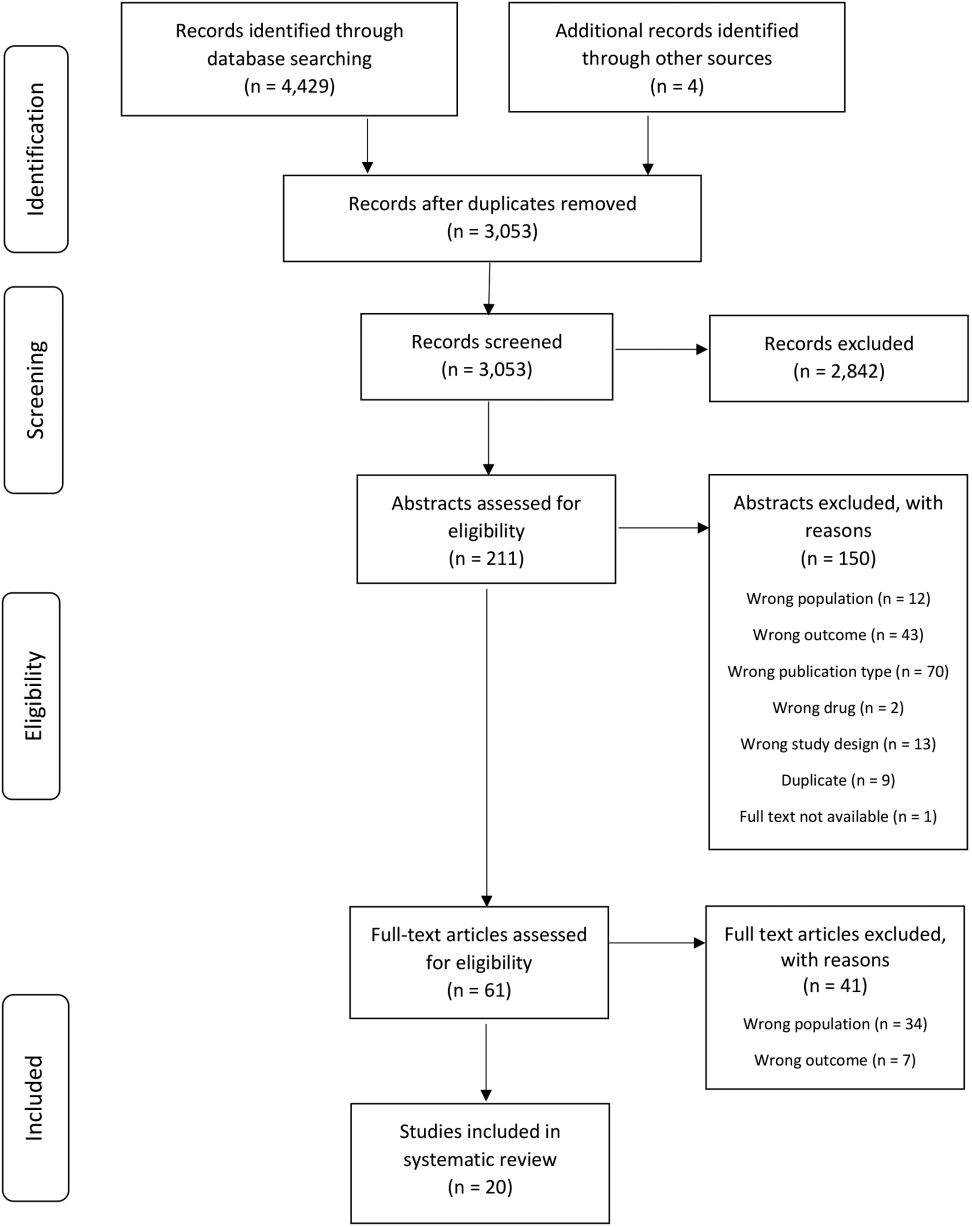
Flow-diagram depicting the selection of included studies.

The searches identified 4429 citations, and 4 titles were identified through hand-searching. After removal of duplicates, 3053 titles and 211 abstracts were assessed for eligibility. Reasons for exclusion at the abstract and full-text stages are provided in Figure 1. A total of 20 articles were included in the review.

### Characteristics of the Included Studies

The systematic review included articles published between 2015 and 2021, comprising a total of 50 470 participants (mean age ranging from 67.9 years^12^ to 83.6 years^13^; 24%^9^ to 71.3%^12^ female, ICrH sustained on OAC ranging from 0% to 100%). Eight articles^13–20^ reported on prospective observational cohorts, 6^9,12,21–24^ on retrospective cohorts, 2^25,26^ on nationwide cohorts, 2^27,28^ meta-analyzed individual-level data from observational studies, 1 reported on the pilot phase of an RCT,^8^ and 1 reported on a Phase 2 trial^7^ (Table S3). Nine studies^8,12–15,23–26^ included patients with an index ICrH (intracerebral, subdural, subarachnoid, or epidural hemorrhages) and 11 studies^7,9,16–22,27,29^ included patients with an index ICH. Eight studies could not be included in meta-analyses, either due to differences in reported outcomes^21,27,28^ or because raw event data were not available.^14,15,17,18,24^

The included articles reported on a mixture of OAC-naive patients and patients who had their index event while on OAC and/ or antiplatelets. The intervention ranged from vitamin-K antagonist (VKA) only,^12,15,22,25,27,28^ nonvitamin K antagonist oral anticoagulants (NOAC) only,^7,16,21,23,26^ a mixture of VKA and NOAC,^9,13,14,17,20,24^ or OAC and/or antiplatelets.^8,18,19^ The most commonly reported outcomes were ischemic stroke and recurrent ICrH. There were variations in how the outcome of ischemic stroke was defined, including cerebral infarct, ischemic stroke, thromboembolic events, major vascular events, and the combined outcome of ischemic event/systemic embolism (Table).

**Table.**
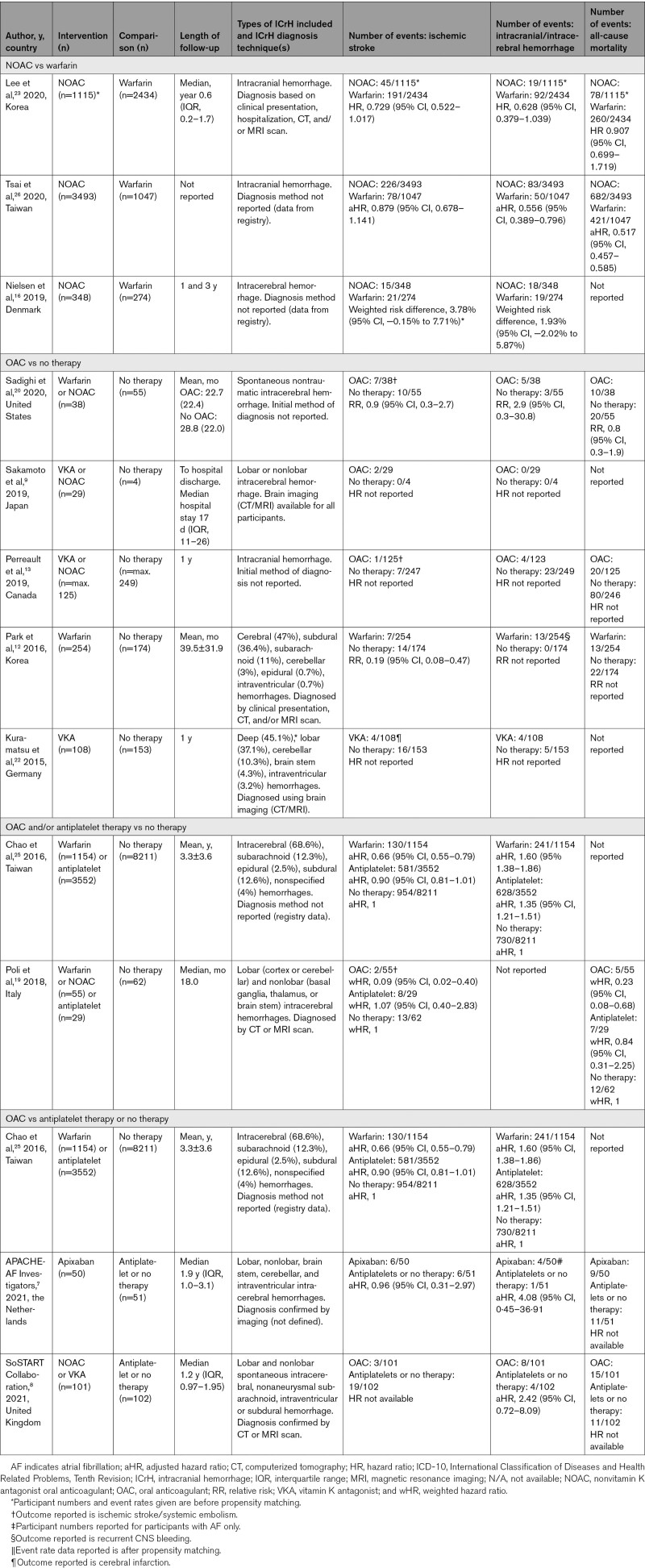
Summary of Event Data by Intervention and Comparator for Articles Included in the Meta-Analyses

### Primary Outcomes

#### Thromboembolic Events

Seventeen articles (n=35 441) reported on the primary outcome of ischemic stroke/systemic embolism or alternative definitions of stroke. Of these, 9 studies reported on ischemic stroke alone,^7–9,16,19,23,25–27^ 5 on ischemic stroke/systemic embolism combined,^13–15,18,20^ 1 on thromboembolic events,^12^ 1 on ischemic stroke combined with transient ischemic attack (TIA),^24^ and 1 on cerebral infarction.^22^ One article included OAC-naive participants,^23^ 8 included participants who sustained an ICrH on OAC^7,15,19,20,22,24,26,27^ and 8 included a combination of both OAC-naive and current OAC users.^8,9,12–14,16,18,25^ Follow-up ranged from a median of 17 days^9^ to a median of 48.6 months.^27^

##### Oral Anticoagulation Versus No Therapy

**Figure 2.**
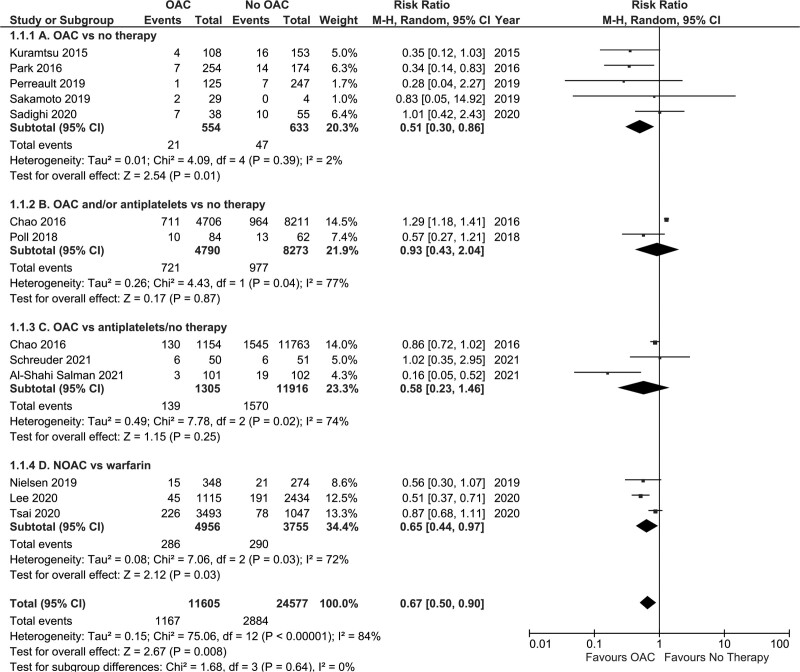
Forest plot depicting the risk of thromboembolic events in patients postintracranial hemorrhages with atrial fibrillation receiving oral anticoagulant (OAC) versus no therapy, OAC and/or antiplatelets versus no therapy, OAC versus antiplatelets/no therapy, or nonvitamin K antagonist oral anticoagulant (NOAC) versus warfarin.

Five studies^9,12,13,20,22^ (n=1187 participants) compared the effect of OAC with no therapy on the risk of thromboembolic events (defined in the included articles as cerebral infarction, ischemic stroke, ischemic stroke/systemic embolism) and were entered into a meta-analysis (Figure 2A), which revealed a significant reduction in thromboembolic events with OAC compared with no therapy (relative risk [RR], 0.51 [95% CI, 0.30–0.86]; *P*=0.01, I^2^=2%).

Three studies could not be entered into a meta-analysis,^15,24,27^ either because raw event data were not available^15,24^ or because the study compared the effect of OAC therapy solely in patients with lobar and nonlobar ICH (with no control group).^27^ Nielsen et al^15^ reported that OAC therapy was associated with a nonsignificant reduction in the rate of ischemic stroke and systemic embolism compared with no therapy (event rate 3.3 versus 8.9 per 100 person-years, adjusted HR, 0.49 [95% CI, 0.24–1.02]). Biffi et al^27^ reported that restarting VKA was associated with a reduced risk of sustaining an ischemic stroke in both lobar (HR, 0.48 [95% CI, 0.25–0.75]; *P*=0.003) and nonlobar (HR, 0.39 [95% CI, 0.21–0.74]; *P*=0.004) ICH patients. Newman et al^24^ reported no statistical significance between the OAC and no therapy groups for the outcome of stroke/TIA (adjusted HR, 0.87 [95% CI, 0.62–121]).

##### Oral Anticoagulation and/or Antiplatelets Versus No Therapy

Three observational studies^18,19,25^ compared OAC and/or antiplatelets with no therapy and reported on the outcome of ischemic events. The study by Pennlert et al^18^ was not included in the meta-analysis due to unavailability of raw event data. The pooled relative risk for the other 2 studies (n=13 063)^19,25^ was RR, 0.93 [95% CI, 0.43–2.04]; *P*=0.87, I^2^=77% (Figure 2B).

##### Oral Anticoagulation Versus Antiplatelets or No Therapy

Three observational studies^14,18,25^ (n=17 287) and 2 RCTs^7,8^ (n=304) compared OAC versus antiplatelet or no therapy. Three studies were included in a meta-analysis^7,8,25^ (n=13 221), which found no significant difference in the risk of thromboembolic events between OAC and antiplatelet or no therapy (RR, 0.58 [95% CI, 0.23–1.46]; *P*=0.25, I^2^=74%; Figure 2C).

Two studies could not be entered into the meta-analysis due to lack of raw event data. Pennlert et al^18^ reported that the cumulative incidence of thromboembolic events 3-years post-index ICH was 6.3% in patients assigned to OAC versus 18.8% in the antiplatelet group and 13.8% in the no therapy group. Nielsen et al^14^ reported that the incidence rate of ischemic stroke/systemic embolism, per 100 person-years, was 5.3 (95% CI, 3.3–8.5) in the OAC group, 10.3 (95% CI, 7.3–14.4) in the antiplatelet group, and 10.4 (95% CI, 8.2–13.1) in the no therapy group.

##### NOAC Versus Warfarin

Three studies (n=8711)^16,23,26^ compared NOAC with warfarin and reported a significant reduction in the risk of thromboembolic events with NOAC compared with warfarin (RR, 0.65 [95% CI, 0.44–0.97]; *P*=0.03, I^2^=72%) but there was considerable heterogeneity (Figure 2D).

#### Recurrent ICrH

Fifteen studies^7–9,12–16,20,22–27^ (n=32 579 patients with AF and ICrH) reported on the outcome of recurrent ICrH. Eight studies^8,13–15,23–26^ included patients with an index ICrH (intracerebral, subdural, subarachnoid, or epidural hemorrhages) and 7 studies^7,9,14,16,20,22,27^ included patients with an index ICH. One study included OAC-naive participants,^23^ 7 studies included participants who sustained an ICrH on OAC,^7,15,20,22,24,26,27^ and 7 studies included a combination of OAC-naive patients and patients who sustained an ICrH on OAC.^8,9,12–14,16,25^ Follow-up ranged from a median of 17 days^9^ to a median 48.6 months.^27^

##### Oral Anticoagulation Versus No Therapy

**Figure 3.**
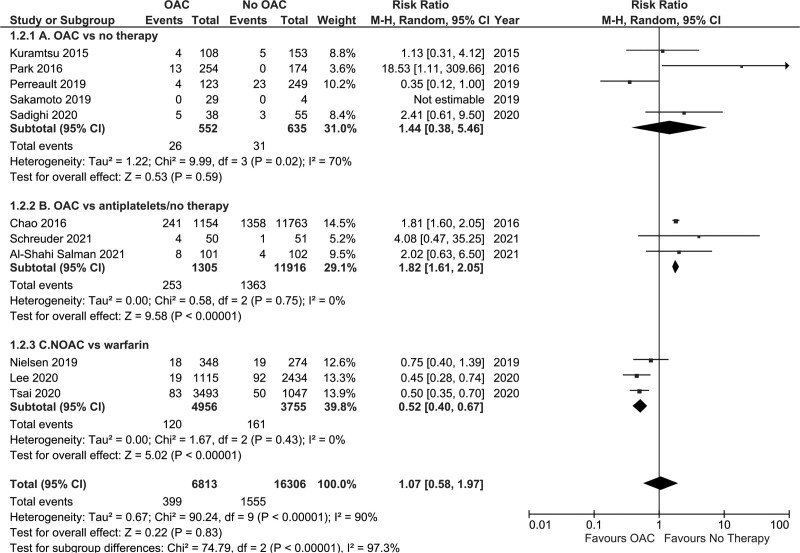
Forest plot depicting the risk of repeat intracranial hemorrhage in patients post-intracerebral hemorrhage with atrial fibrillation receiving oral anticoagulant (OAC) versus no therapy, OAC versus antiplatelets/no therapy, or nonvitamin-K antagonist oral anticoagulant (NOAC) versus warfarin.

Five observational studies (n=1187)^9,12,13,20,22^ compared OAC versus no therapy on the risk of sustaining a recurrent ICrH and the pooled estimate revealed no statistically significant difference (RR, 1.44 [95% CI, 0.38–5.46]; *P*=0.59, I^2^=70%; Figure 3A).

Three observational studies could not be entered into a meta-analysis.^15,24,27^ Biffi et al^27^ reported that VKA resumption was associated with a nonsignificant increase in the risk of sustaining an ICH (lobar HR, 1.21 [95% CI, 0.86–1.70]; *P*=0.27; nonlobar HR, 1.10 [95% CI, 0.94–1.28]; *P*=0.23). Nielsen et al^15^ reported that warfarin treatment was associated with a nonsignificant increase in the risk of recurrent ICrH (adjusted HR, 1.31, [95% CI, 0.68–2.50]). Newman et al^24^ reported an association between OAC therapy (VKA or NOAC) post-ICrH and a reduction in the risk of recurrent ICrH (incidence 3.29 versus 5.80 events per 100 patient years, adjusted HR=0.62 [95% CI, 0.41–0.95]).

##### Oral Anticoagulation Versus Antiplatelets or No Therapy

Two RCTs^7,8^ (n=304) and 2 observational studies^14,25^ (n=14 669) compared OAC versus antiplatelet therapy or no therapy. Three studies^7,8,25^ (n=13 221) were entered into a meta-analysis; OAC was associated with a higher risk of recurrent ICrH versus antiplatelet or no therapy (RR, 1.82 [95% CI, 1.61–2.05]; *P*<0.01, I2=0%; Figure 3B).

Nielsen et al.^14^ reported that the incidence rate of recurrent ICrH per 100 person-years, was 8.0 (95% CI, 5.4–11.8) in the OAC group, 5.3 (95% CI, 3.3–8.4) in the antiplatelet group, and 8.6 (95% CI, 6.6–11.2) in the no therapy group.

##### NOAC Versus Warfarin

Three studies (n=8711)^16,23,26^ compared the effect of NOAC versus warfarin on recurrent ICrH with the pooled relative risk demonstrating that NOAC significantly reduced risk of recurrent ICrH compared with warfarin (RR, 0.52 [95% CI, 0.40–0.67]; *P*<0.00001, I^2^=0%; Figure 3C).

### Secondary Outcomes

#### All-Cause Mortality

Five observational studies (n=11 456)^12,13,20,23,26^ and 2 RCTs^7,8^ (n=304) reported on all-cause mortality. Three studies included patients who had sustained their index ICrH on OAC,^7,20,26^ 1 study included OAC-naive patients,^23^ and another 3 included a mixture of OAC-naive patients and patients who sustained their ICrH on OAC.^8,12,13^ Two studies included patients who sustained an index ICH^7,20^ and 5 articles included patients who sustained an index ICrH.^8,12,13,23,26^ The longest follow-up was median 39.9 months.^12^

##### Oral Anticoagulation Versus No Therapy

**Figure 4.**
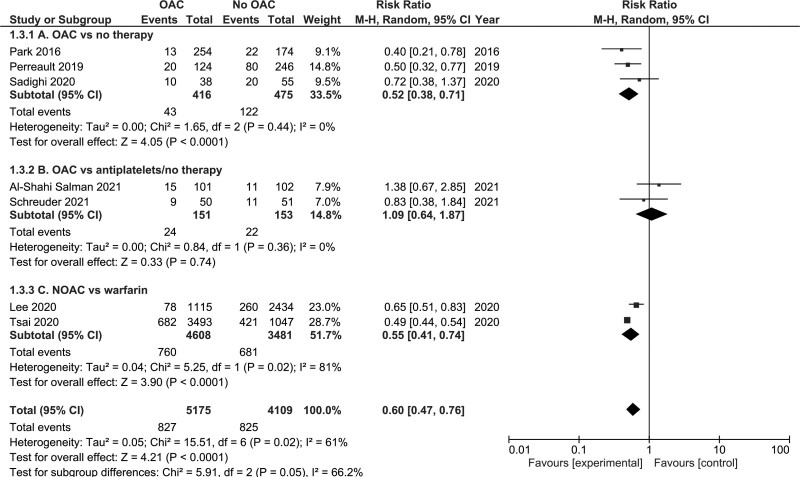
Forest plot depicting the risk of all-cause mortality in patients postintracranial hemorrhage with atrial fibrillation receiving oral anticoagulation (OAC) versus no therapy, OAC versus antiplatelets/no therapy, or nonvitamin K antagonist oral anticoagulant (NOAC) versus warfarin.

Three studies (n=891)^12,13,20^ examined the impact of OAC versus no therapy on all-cause mortality and reported a significant reduction in death associated with OAC (RR, 0.52 [95% CI, 0.38–0.71]; *P*<0.01, I^2^=0%; Figure 4A).

##### Oral Anticoagulation Versus Antiplatelets or No Therapy

Two RCTs (n=304) examined the impact of OAC versus antiplatelets or no therapy on all-cause mortality and were entered into a meta-analysis, which was not statistically significant (RR, 1.09 [95% CI, 0.64–1.87]; *P*=0.74, I^2^=0%; Figure 4B).

##### NOAC Versus Warfarin

Two studies (n=8089)^23,26^ examined the impact of NOAC versus warfarin on the risk of all-cause mortality and the pooled estimate demonstrated that NOACs were significantly associated with a reduced risk of all-cause mortality (RR, 0.55 [95% CI, 0.41–0.74]; *P*<0.00001, I^2^=81%; Figure 4C).

##### Subgroup and Sensitivity Analyses

Subgroup and sensitivity analyses are provided in Table S4. OAC therapy significantly reduced the risk of thromboembolic events at 1 year follow-up (RR, 0.34 [95% CI, 0.13–0.87], I^2^=0%) but not at > 1 year (RR, 0.59 [95% CI, 0.20–1.72], I^2^=66%). The risk of sustaining a recurrent ICrH did not differ by follow-up time. Examining only the RCT data^7,8^ (n=304), no difference was found in the risk of ischemic stroke (RR, 0.41 [95% CI, 0.06–2.64], I^2^=82%) or recurrent ICH (RR, 2.37 [95% CI, 0.85–6.62], I^2^=0%) when comparing OAC with antiplatelet or no therapy.

#### Risk of Bias Assessment

The overall risk of bias assessment for all included studies is presented in Figures S1 and S2. The categories addressing participant selection, incomplete outcome data, and selective outcome reporting among observational studies were assessed as having the lowest risk of bias.

## Discussion

This systematic review included 20 studies (n=50 470, 304 were enrolled in RCTs) and updates previously published reviews.^29–31^ Our main findings are that OAC significantly reduced the risk of thromboembolic events and all-cause mortality in patients with AF and ICrH, without significantly increasing the risk of recurrent ICrH. Second, NOACs were associated with a lower risk of thromboembolic events and recurrent ICrH than warfarin.

### Oral Anticoagulation for Stroke Prevention

This PRISMA-compliant systematic review found that OAC therapy significantly reduced the risk of an ischemic stroke in patients with AF and a history of ICrH when compared with no therapy (RR, 0.51 [95% CI, 0.30–0.86]; *P*=0.01). Previous meta-analyses that investigated the effect of restarting OAC post-ICrH reported that OAC generally and VKA specifically were associated with a reduction in thromboembolic events.^29,30,32^ The current review also found that NOAC therapy is more effective at preventing thromboembolic events than warfarin (RR, 0.65 [95% CI, 0.44–0.97]). Although trials comparing NOAC and warfarin largely excluded patients with ICrH, and findings in this review are based on observational data, there is evidence to suggest that NOACs are more effective at preventing thromboembolic events than warfarin.^33^ However, 2 recently completed RCTs found that restarting OAC was not associated with a significant decrease in the risk of thromboembolic events in patients with AF and a history of ICH or ICrH.^7,8^

### Oral Anticoagulation and All-Cause Mortality

The significant reduction in all-cause mortality among patients with AF who received OAC therapy following ICrH compared with those who received no therapy supports the use of OAC in the post-ICrH population with AF. This finding is confirmed by a previous meta-analysis that examined OAC resumption in patients who sustained an OAC-associated ICrH.^32^ The main limitation with assessing the impact of OAC therapy following ICrH is confounding by indication, as studies have shown differences between those who did and those who did not receive OAC post-ICrH.^34–36^ Two studies also reported that patients exposed to OAC at the time of their ICrH were less likely to restart OAC post-ICrH.^12,13^ It is possible that OAC is associated with improved survival in AF patients post-ICrH as OAC is more likely to be prescribed to those who are more likely to survive. However, OAC use post-ICrH has been shown to be associated with improved functional outcomes among patients with poor functional status (modified Rankin Scale score >3) at hospital discharge.^27,28^

### Oral Anticoagulation and the Risk of Recurrent ICrH

The current review found that OAC therapy was not associated with a statistically significantly increased risk of recurrent ICrH (RR, 1.44 [95% CI, 0.38–5.46]; *P*=0.59). Of the 10 studies examining the association between OAC and/or antiplatelet therapy and the risk of recurrent ICrH, 2 studies^12,25^ reported a significant increase in the risk of an ICrH (defined in 1 article as CNS bleeding), 2 studies^13,24^ reported a reduction in the risk of repeat ICrH, and 6^7,8,14,20,22,27^ studies reported no significant difference in the risk of recurrent ICrH. There was heterogeneity in the type of OAC therapy used and the baseline characteristics of the patients who were commenced on OAC therapy. Furthermore, there was considerable heterogeneity in the participant cohorts and follow-up periods reported, and an unclear risk of bias regarding measurement of participants’ exposure to OAC therapy in all but one of the included articles.

From the patient’s perspective, the key attribute of OAC therapy is stroke prevention, although risk of bleeding is the second most important attribute when choosing OAC.^37^ However, patients report variability in the number of acceptable bleeds associated with OAC therapy and considerable differences in the percentage of patients who were not willing to consider OAC therapy.^38^ The study by Chao et al^25^ reported that patients who survive an ICrH are at increased risk of repeat ICrH regardless of whether they receive OAC therapy post-ICrH or not, and that OAC therapy with VKA post-ICrH ought to be reserved for patients with CHA_2_DS_2_-VASc ≥6. NOACs may alleviate some concerns about OAC-related ICrH because apixaban and dabigatran have been shown to be associated with reduced risk of major bleeding when compared with warfarin,^39^ and NOACs have reversal agents which may prevent exacerbating the ICrH. However, results from APACHE-AF^7^ show that there were more recurrent ICH in the apixaban group than in the antiplatelet or no therapy group (8% versus 2%), although this difference was not significant (adjusted hazard ratio, 4.08 [95% CI, 0.45–36.91]). Furthermore, SoSTART^8^ reported that OAC could not be considered noninferior to no therapy due to the increased risk of mortality and recurrent ICrH.

### Strengths and Limitations of This Review

Several bibliographic databases were searched to ensure that all contemporary relevant literature was captured, and 2 authors independently selected the included studies and extracted the data. Sensitivity analyses and subgroup analyses were undertaken.

The primary limitation of this review is that most are observational cohort studies. The included studies were heterogeneous, both clinically and methodologically, with most reporting on both intracerebral and other types of ICrH combined. This is a limitation since intracerebral (or parenchymal) hemorrhage is associated with a higher intrinsic risk of thrombotic events than subarachnoid hemorrhage. In addition, not all studies could be included in the meta-analyses due to unavailable data or differences in reported outcomes. Finally, it was difficult to accurately assess the measurement of participants’ exposure to OAC therapy, since several studies were retrospective in design and utilized patient data from large databases. Therefore, the results of this systematic review should be interpreted with caution. Several RCTs addressing the efficacy and safety of OAC for stroke prevention in patients with AF who have survived an ICH or ICrH are ongoing.^40–45^ The findings reported by these RCTs will be critical to confirm or refute the findings of this review.

## Conclusions

OAC use after ICrH in patients with AF significantly reduces the risk of thromboembolic events and all-cause mortality, without significantly increasing the risk of recurrent ICrH. NOACs are preferable to warfarin as they are associated with preventing thromboembolic events with a lower risk of recurrent ICrH. Nevertheless, the available evidence is mostly observational, with considerable clinical and methodological heterogeneity, including differences in intervention, comparators, outcomes, and follow-up time. Thus, further evidence from ongoing RCTs is urgently needed to corroborate these findings.

## Article Information

### Acknowledgments

The authors would like to thank Dr Yuki Sakamoto and colleagues for their provision of supplementary data to accompany the article included in the review.

### Sources of Funding

This project has received funding from the European Union’s Horizon 2020 research and innovation programme under grant agreement No 754517.

### Disclosures

E. Ivany is a Research Associate on the PRESTIGE-AF study, which has received funding from the European Union’s Horizon 2020 research and innovation programme under Grant Agreement No. 754517. Dr Lane has received investigator-initiated educational grants from Bristol-Myers Squibb (BMS), has been a speaker for Bayer, Boehringer Ingeheim, and BMS/Pfizer and has consulted for BMS, and Boehringer Ingelheim. Dr Lane is a co-investigator on the PRESTIGE-AF study, which has received funding from the European Union’s Horizon 2020 research and innovation programme under Grant Agreement No. 754517. Dr Lip is a consultant and speaker for BMS/Pfizer, Boehringer Ingelheim, and Daiichi-Sankyo. No fees are received personally. Dr Lip is a co-investigator on the PRESTIGE-AF study, which has received funding from the European Union’s Horizon 2020 research and innovation programme under Grant Agreement No. 754517. Dr Lip was part of the steering committee for the SoSTART trial. Dr Werring received honoraria for consulting or lecturing from Alexion, Alnylam, Bayer, Novo Nordisk, and Portola. The other authors report no conflicts.

### Supplemental Material

Tables S1–S4

Figures S1–S2

MOOSE and PRISMA Checklists

## Supplementary Material


