# Supplementary material for: Effectiveness and Safety of Antithrombotic Medication in Patients With Atrial Fibrillation and Intracranial Hemorrhage: Systematic Review and Meta-Analysis
Source: Stroke. 2022 Jul 8;53(10):3035–46. doi: 10.1161/STROKEAHA.122.038752 (PMC9674450; doi:10.1161/STROKEAHA.122.038752)
Supplement: Supplementary file 1 [file str-53-3035-s001.pdf]

SUPPLEMENTAL MATERIAL

Supplementary tables S1-S4

Supplementary figures S1-S2

MOOSE checklist for meta-analyses of observational studies

PRISMA checklist

Supplementary Table S1: Definition of terms

Supplementary Table S2: Search terms used during data searching

(CENTRAL: Cochrane Central Register of Controlled Trials; CINAHL: Cumulative Index to Nursing and Allied Health)

Supplementary Table S3: Summary characteristics of included studies

(AF: atrial fibrillation; CHA_2DS_2-VASc: congestive heart failure, hypertension, age >75, diabetes mellitus, prior stroke, vascular disease, age 65-74, sex; ICH: intracerebral haemorrhage; IQR: inter-quartile range; NOAC: non- vitamin K antagonist oral anticoagulant; OAC: oral anticoagulation; SD: standard deviation; VKA: vitamin-K antagonist)

Supplementary Table S4: Results of sub-group and sensitivity analyses

(APACHE-AF: Apixaban After Anticoagulation-associated Intracerebral Haemorrhage in Patients with Atrial Fibrillation; CI: confidence interval; ICrH: intracranial haemorrhage; RR: risk ratio; SoSTART: Start or STop Anticoagulants Randomised Trial)

Supplementary Table S1: Definition of Terms

Term	Definition*
Atrial fibrillation	A disease characterised by the chaotic generation of electrical impulses in the atria, leading to an irregular heart rhythm and an increased risk of thromboembolic events due to formation of blood clots.
Central nervous system bleeding	Bleeding that occurs within the brain or spinal cord.
Cerebral infarct	Prolonged ischaemia that results in focal necrosis of brain tissue.
Cerebral micro-bleed	Small focal intracerebral haemorrhages.
CHA_2DS_2-VASc	A risk scoring tool that can be used to predict the risk of a thromboembolic event in an individual with atrial fibrillation. The tool considers the following risk factors: congestive heart failure, hypertension, age >75, diabetes mellitus, prior stroke, vascular disease, age 65-74, sex.
Functional status	An individual's ability to perform basic activities of daily living.
International normalised ratio	A laboratory measurement used to determine the effects of oral anticoagulants on the body's blood clotting.
Intracerebral haemorrhage	Bleeding that occurs within the brain parenchyma. Includes cerebellar, brain steam, and deep haemorrhages.
Intracranial haemorrhage	Bleeding that occurs within the skull. Includes intracerebral, subdural, subarachnoid, and epidural haemorrhages.
Ischaemic stroke	A stroke caused by blockage of blood supply in the brain.
Major vascular event	An injury that affects the vascular system, such as ischaemic stroke, myocardial infarction, sudden cardiac death.
Modified Rankin scale	A tool that assesses disability in an individual who has suffered a stroke.
Oral anticoagulation	Oral medication that prevents coagulation of the blood.
Systemic embolism	Presence of emboli (clots) in the arterial circulation.
Thromboembolic event	The formation of a blood clot that partially or fully obstructs venous or arterial blood flow. This includes conditions such as ischaemic stroke, systemic embolism, transient ischaemic attack).
Time in therapeutic range	The percentage of time in which a patient's international normalised ratio remains in the target range.
Transient ischaemic attack	Temporary disruption of blood supply in the brain.

*Other definitions as used by the included papers were also accepted by the systematic review authors.

Supplementary Table S2: Search terms used during data searching

MEDLINE and EMBASE via Ovid	CINAHL via EbscoHost	CENTRAL
1.atrial fibrillation.mp	1. (MM “Atrial Fibrillation”)	1. MESH descriptor Atrial Fibrillation explode all trees
2.exp atrial fibrillation/	2. AF or atrial fibrillation	2. MESH descriptor Fibrinolytic Agents explode all trees
3. (AF or AFib).ab	3. (MM “Hematologic agents”)	3. MESH descriptor Thrombolytic Therapy explode all trees
4.exp cerebral hemorrhage/	4. (MM “Anticoagulants”)	4. MESH descriptor Cerebral Hemorrhage explode all trees
5. cerebral hemorrhage.mp	5. (MM “Thrombolytic Therapy)	5. atrial fibrillation
6. (intracerebral adj6 hemorrhage).ab	6. antithrombotic or anticoagulant	6. antithrombotic
7. (intracerebral adj6 haemorrhage).ab	7. Apixaban OR edoxaban OR rivaroxaban OR dabigatran	7. anticoagulant
8. (cerebral adj6 bleed*).ab	8. Novel oral anticoagulant OR NOAC	8. antiplatelet
9. (intracerebral adj6 bleed*).ab	9. Non-vitamin K antagonist oral anticoagulant	9. intracerebral hemorrhage
10. (spontaneous adj6 intracerebral adj6 hemorrhage).ab	10. Direct oral anticoagulant OR DOAC11. Factor Xa inhibitor	10. #1 OR #5
11 (spontaneous adj6 intracerebral adj6 haemorrhage).ab	12. antiplatelet therapy	11. #2 OR #3 OR #6
12. (spontaneous adj6 intracerebral adj6 bleed*).ab	13. vitamin K antagonist OR VKA	12. #6 AND #7 AND #8
13. (non-traumatic adj6 intracerebral adj6 hemorrhage).ab	14. thrombolytic therapy in stroke	13. #11 OR #12
14. (non-traumatic adj6 intracerebral adj6 haemorrhage).ab	15. intracerebral hemorrhage	14. #4 OR #9
15. (non-traumatic adj6 intracerebral adj6 bleed*).ab	16. intracerebral hemorrhage stroke	15. #10 AND #13 AND #14
16. (hemorrhagic adj6 stroke).ab	17. intracerebral stroke	
17. (haemorrhagic adj6 stroke).ab	18. 1 OR 2	
18. exp Anticoagulants/	19. 3 OR 4 OR 5 OR 6	
19. anticoagulants.mp	20. 7 OR 8 OR 9 OR 10 OR 11 OR 12 OR 13 OR 14	
20. (anticoagula$ OR oral anticoagula$).ab	21. 15 OR 16 OR 17	
21. (direct adj6 oral adj6 anticoagula$).ab	22. 19 OR 20	
22. (novel adj6 oral adj6 anticoagula$).ab	23 18 AND 21 AND 22	
23. (non-vitamin aj6 K adj6 inhibitor adj6 oral adj6 anticoagula$).ab	24. 23 limiters - Publication Year: 2000-2020	
24. (NOAC OR DOAC).ab		
25. (rivaroxaban OR dabigatran OR edoxaban OR apixaban).ab		
26. 1 OR 2 OR 3		
27. 4 OR 5 OR 6 OR 7 OR 8 OR 9 OR 10 OR 11 OR 12 OR 13 OR 14 OR 15 OR 16 OR 17		
28. 18 OR 19 OR 20 OR 21 OR 22 OR 23 OR 24 OR 25		
29. 26 AND 27 AND 28		
30. limit 29 to yr=”2000-Current”		
31. limit 30 to (adaptive clinical trial or clinical study or clinical trial, all or clinical trial or comparative study or controlled clinical trial or equivalence trial or evaluation study or multicenter study or observational study or pragmatic clinical trial or randomized controlled trial)		

Supplementary Table S3: Summary characteristics of included studies

First author, year, country	Study design	Key inclusion criteria	N	Female (%)	Mean age (years) [SD]	Mean CHA_2DS_2-VASc [SD]	Intervention	Comparison	Primary outcomes	Follow-up length	OAC at time of ICH (%)	Period of data collection
**OAC vs no therapy**												
Sadighi 2020, USA[20]	Observational cohort	Non-traumatic intracerebral haemorrhage, non-valvular and valvular AF (incl. prosthetic heart valves) and atrial flutter.	93	45.7%	76.2 [10.3]	Not reported	Warfarin or NOAC	No therapy	Rate of recurrent ICH, ischaemic stroke/ systemic symbolism, death	Mean, months **OAC** 22.7 [22.4] **No OAC** 28.8 [22.0]	100%	Patients hospitalised in the period 2010-2017
Newman 2020, USA[24]	Retrospective cohort	Non-valvular AF diagnosed prior to index event, OAC-associated intracranial haemorrhage, filed OAC prescription within 6 months of index event.	1,502	56.3%	Not reported	Not reported	VKA or NOAC	No therapy	Composite ischaemic stroke/TIA, recurrent ICH, composite ischaemic stroke/ TIA/ICH, all-cause mortality, thromboembolism	Average 780 days	100%	1/1/2020 – 31/10/2016
Perreault 2019, Canada[13]	Observational cohort	Aged ≥65, non-valvular AF, intracranial haemorrhage on or off OAC, not on antiplatelets or dual therapy	683	**OAC** 53.9% **No OAC** 52.7%	**OAC** 81.7 [5.8] **No OAC** 83.6 [5.8]	Not reported	VKA or NOAC	No therapy	Ischaemic stroke/ systemic embolism, all-cause mortality, repeat ICH, major bleeding	Median 12 months	[1]**OAC group** 89.6% **No treatment** 69%	1/1/1995 – 31/12/2015
Sakamoto, 2019, Japan[9]	Retrospective cohort	Acute spontaneous intracerebral haemorrhage, anticoagulated for AF or deep vein thrombosis, diagnosed during stroke unit stay	236 (33 AF)	**Cohort** 34% **AF only** 24%	Median, IQR **Cohort** 69 [61-79] **AF only** 72 [65-82]	**AF only** 5.24 [1.62]	VKA or NOAC	No therapy	Clinical characteristics of patients in OAC-restart vs. OAC non-restart groups and in OAC-indicated and OAC restart vs OAC-indicated and OAC non-restart groups, no. of days from ICH to OAC therapy,	To hospital discharge. Median hospital stay 17 days [IQR 11-26]	[2]**OAC group** 61% **No treatment** 50%	Consecutive patient admissions September 2014 – March 2017
Kato, 2019, Japan[21]	Retrospective cohort	Non-valvular AF, acute spontaneous intracerebral haemorrhage, on NOAC at time of ICH	43	**OAC** 47% **No OAC** 29%	**OAC** 74.1 **No OAC** 72.4	Median [IQR] 4 [3-5]	NOAC	No therapy	Timing of NOAC resumption; factors that influence NOAC resumption	**OAC** 31 days **No OAC** 32 days	100%	Patient admissions March 2014 – February 2018
Nielsen, 2017, Denmark[15]	Observational cohort	Non-traumatic or traumatic intracranial haemorrhage, prior diagnosis of AF, prior treatment with warfarin	2,415	38.7%	77.1 [9.1]	3.9 [1.7]	Warfarin	No therapy	Composite outcome of ischaemic stroke and systemic embolism, recurrent ICH.	Median 279 days	100%	1/1/1998 – 28/2/2016

Park, 2016, Korea[12]	Retrospective cohort	AF (incl. valvular, prosthetic valves, PE, DVT), prior intracranial haemorrhage, requiring OAC, spontaneous or traumatic ICH	428	**OAC**<br>62.2%<br>**No OAC**<br>71.3%	**OAC**<br>67.9<br>(11.1)<br>**No OAC**<br>69.1<br>[10.8]	**OAC**<br>3.35 [1.7]<br>**No OAC**<br>3.16 [1.73]	Warfarin	No therapy	Thromboembolic (incl. ischaemic stroke and systemic embolisms) and major bleeding events; composite endpoint	Mean 39.5 ± 31.9 months	[1]**OAC group**<br>65%<br>**No OAC group**<br>39.1%	Patient enrolment 1/1/2009 – 31/12/2013
Murphy, 2018, USA/ Germany[28]	Meta-analysis of individual-level data	Primary and first intracerebral haemorrhage, non-valvular AF requiring OAC, on OAC at time of ICH, no prior functional impairment, no ICH/ ischaemic stroke at 1-year follow up	941	**RETRACE**<br>39%<br>**MGH**<br>40%<br>**ERICH**<br>32%	**RETRACE** 73.6<br>[7.8]<br>**MGH** 72.1<br>[9.3]<br>**ERICH**<br>71.2<br>[11.3]	Median [IQR]<br>**RETRACE**<br>5 [4-6]<br>**MGH**<br>5 [4-6]<br>**ERICH**<br>5 [4-6]	VKA	No therapy	Recovery in functional status at 1-year post index ICH	3, 6, 12 months post index ICH	100%	Study dates<br>RETRACE<br>2006 – 2010<br>ERICH<br>2011 – 2015<br>MGH<br>1994 – ongoing in 2018
Biffi, 2017, USA/ Germany[27]	Meta-analysis of individual-level data	First acute intracerebral haemorrhage, non-traumatic ICH, on OAC at time of ICH (for stroke prevention in AF, VKA with INR >1.5), potential for OAC resumption.	1,012	**RETRACE**<br>39%<br>**MGH**<br>40%<br>**ERICH**<br>32%	**RETRACE** 73.6<br>[7.8]<br>**MGH** 72.1<br>[9.3]<br>**ERICH**<br>71.2<br>[11.3]	Median [IQR]<br>**RETRACE**<br>5 [4-6]<br>**MGH**<br>5 [4-6]<br>**ERICH**<br>5 [4-6]	VKA	No therapy	Association between OAC resumption and 90 day-mortality. Functional outcomes	Median, months.<br>**Lobar ICH**<br>48.6 [IQR 33.9-65.4]<br>**Non-lobar ICH** - 46.7 [IQR 32.6 – 63.4]	100%	Study dates<br>RETRACE<br>2006 – 2010<br>ERICH<br>2011 – 2015<br>MGH<br>1994 – ongoing in 2017
Kuramatsu, 2015, Germany[22]	Retrospective cohort	Spontaneous intracerebral haemorrhage, on VKA on admission, INR on admission >1.5.	719 (566 AF)	**OAC**<br>32.7%<br>**No OAC**<br>39.9%	**OAC**<br>72.9 [7.7]<br>**No OAC**<br>75.5 [7.8]	Median $CHADS_2$ [IQR}<br>**OAC** 2 [1-3]<br>**No OAC** 2 [2-3]	VKA	No therapy	OAC cohort: ischaemic or non-ischaemic stroke, haemorrhagic or extra-cerebral bleeding	One year	100%	RETRACE 2006 – 2010
**OAC vs antiplatelets or no therapy**												
SoSTART Collaboration, 2021, UK[8]	Randomised controlled trial (pilot phase)	Diagnosed AF, survived >24 hours post spontaneous symptomatic intracranial haemorrhage, CHA_2DS_2VASc >2.	203	**OAC**<br>**39%**<br>**No OAC**<br>**36%**	Median [IQR]<br>**OAC**<br>79 [74-85]<br>**No OAC**<br>79 [74-84]	Median [IQR]<br>4 [3-5]	NOAC or VKA	Antiplatelet or no therapy	Recurrent symptomatic spontaneous intracranial haemorrhage	Median 1.2 years [IQR 0.97-1.95]	*OAC use prior to ICH*<br>**OAC**<br>83%<br>**No OAC**<br>84%	29/3/20018-27/2/2020
APACHE-AF Investigators, 2021, The Netherlands[7]	Randomised controlled trial (phase 2)	Spontaneous intracerebral haemorrhage while on OAC, CHA_2DS_2-VASc ≥2, mRS ≤4.	101	46%	Median [IQR]<br>78 [73-83]	Median [IQR]<br>4 [3-5]	Apixaban	Antiplatelet or no therapy	Non-fatal stroke (ischaemic or intracranial) or vascular death.	Median 1.9 years [IQR 1.0-3.1]	100%	15/01/2015-06/06/2020
Chao,2016, Taiwan[25]	Nationwide cohort	Newly diagnosed AF, with or without prior intracranial	**With ICH**<br>12,917	**With ICH**<br>43%	74.7<br>[11.2]	Median [IQR]<br>**With ICH**	Warfarin	Antiplatelet or no therapy	Clinical (ischaemic stroke) and safety (ICH	Mean 3.3 ± 3.6 years	0.5%	1/1/1996 – 31/12/2011

		haemorrhage, some patients had a diagnosis of head injury.	**Without ICH** 149,618	**Without ICH** 51%		6.0 [5-7] **Without ICH** 4.0 [3-5]			requiring admission to ITU) end points			
Pennlert, 2015, Sweden[17]	Observational cohort	First intracerebral haemorrhage, AF or other indication for OAC post ICH	14,045	At index ICH 43.6%	At index ICH 71.5	Median 4	NOAC or warfarin	Antiplatelet	Timing and predictors of, and extent to which antithrombotics are used in ICH survivors	Mean, days **OAC** 985 **APT** 778	[1]10.4%	1/7/2015 – 31/12/2011
Nielsen, 2015, Denmark[14]	Observational cohort	Non-valvular AF diagnosed prior to ICH, intracranial haemorrhage requiring hospitalisation	1,752	38%	Median 78.0 [IQR 71.0-83.0]	Not reported	NOAC or VKA	Antiplatelet or no therapy	Combined end point of Ischaemic stroke/systemic embolism and all-cause mortality	Data at 1 year	VKA 65% NOAC 2%	Patients diagnosed with AF 1/1/1997 – 31/12/2013
**OAC and/or antiplatelets vs no therapy**												
Poli, 2018, Italy[19]	Observational cohort	Valvular AF or atrial flutter, non-traumatic intracerebral haemorrhage, on OAC at time of ICH	244	41.4%	76.3 [9.7]	Not reported	Warfarin or NOAC or antiplatelet	No therapy	Composite all-cause mortality and ischaemic stroke/ systemic embolism	Median, months 18.0	100%	Consecutive patient admissions 1/1/2002 – 31/7/2013
Pennlert, 2017, Sweden[18]	Observational cohort	First intracerebral haemorrhage, non-traumatic ICH, concomitant AF	2,618	40.7%	78.0 [9.1]	4.13	VKA or NOAC or antiplatelet	No therapy	Thrombotic events, haemorrhagic events, thrombotic and haemorrhagic events combined	Median, years 1.7	47.3%	1/7/2005 – 31/12/2011
**NOAC vs warfarin**												
Lee, 2020, Korea[23]	Retrospective cohort	Non-valvular AF, spontaneous intracranial haemorrhage, not on OAC at time of ICH	5,712	43.1%	72.4 [10.0]	4.0 [1.6]	NOAC	Warfarin	Occurrence of ischaemic stroke, ICH, and composite outcome (ICH + IS)	Median 0.6 year [IQR 0.2-1.7]	0%	January 2010 – April 2018
Tsai, 2020, Taiwan[26]	Nationwide cohort	Newly diagnosed AF, prior intracranial haemorrhage, on or off OAC at time of ICH	4,540	41.6%	76.0 [10.5]	5.5. [1.67]	NOAC	Warfarin	Annual risk of clinical (all-cause mortality, ischaemic stroke) and safety (ICH, major bleeding, adverse events) end points	Not reported	100%	1/1/2012-31/12/2016
Nielsen, 2019, Denmark[16]	Observational cohort	Primary spontaneous intracerebral haemorrhage, non-valvular AF diagnosed prior to ICH, claimed OAC prescription during data collection period	622	39.1%	76.1 [9.2]	4.4 [1.7]	NOAC	Warfarin	Effectiveness (ischaemic stroke) and safety (intracerebral haemorrhage) outcome	1 and 3 years	45.2%	January 2003 – April 2017

AF: atrial fibrillation; CHA_2DS_2-VASc: congestive heart failure, hypertension, age >75, diabetes mellitus, prior stroke, vascular disease, age 65-74, sex; ICH: intracerebral haemorrhage; IQR: inter-quartile range; NOAC: non- vitamin K antagonist oral anticoagulant; OAC: oral anticoagulation; SD: standard deviation; VKA: vitamin-K antagonist

Supplementary Table S4: Results of sub-group and sensitivity analyses

Intervention	Comparator	Outcome	Sub-group/sensitivity analyses	Included studies	Participants (n)	Result
Oral anticoagulation	No therapy	Thromboembolic events	1 year follow-up	Kuramatsu (2015)[22]; Perreault (2019)[13]	633	RR 0.34, 95% CI 0.13-0.87, p=0.03, I^2=0%
Oral anticoagulation	No therapy	Thromboembolic events	>1 year follow-up	Park (2016)[12]; Sadighi (2020)[20]	521	RR 0.59, 95% CI 0.20-1.72, p=0.33, I^2=66%
Oral anticoagulation	No therapy	Thromboembolic events	Ischaemic stroke/systemic embolism only	Perreault (2019)[13]; Sadighi (2020)[20]	465	RR 0.75, 95% CI 0.25-2.21, p=0.61, I^2=24%
Oral anticoagulation	Antiplatelets/ no therapy	Ischaemic stroke	Data from randomised controlled trials only	SoSTART, 2021[8]; APACHE-AF, 2021[7]	304	RR 0.41, 95% CI 0.06-2.64, p=0.35, I^2=82%
Oral anticoagulation	No therapy	Recurrent ICrH	1 year follow-up	Kuramatsu (2015)[22]; Perreault (2019)[13]	633	RR 0.59, 95% CI 0.19-1.87, p=0.37, I^2=38%
Oral anticoagulation	No therapy	Recurrent ICrH	>1 year follow-up	Park (2016)[12]; Sadighi (2020)[20]	521	RR 4.97, 95% CI 0.56-44.17, p = 0.15, I^2 = 53%
Oral anticoagulation	Antiplatelets/ no therapy	Recurrent ICrH	Data from randomised controlled trials only	SoSTART (2021)[8]; APACHE-AF (2021)[7]	304	RR 2.37, 95% CI 0.85-6.62, p=0.10, I^2=0%

APACHE-AF: Apixaban After Anticoagulation-associated Intracerebral Haemorrhage in Patients with Atrial Fibrillation; CI: confidence interval; ICrH: intracranial haemorrhage; RR: risk ratio; SoSTART: Start or STop Anticoagulants Randomised Trial

Supplementary Figure S1: Risk of bias assessment for the included observational studies by category (A) and by study (B)

Supplementary Figure S2: Risk of bias assessment for the included randomised controlled trial

Supplementary Figure S1: Risk of bias assessment for the included observational studies

A. Risk of bias assessment* by category

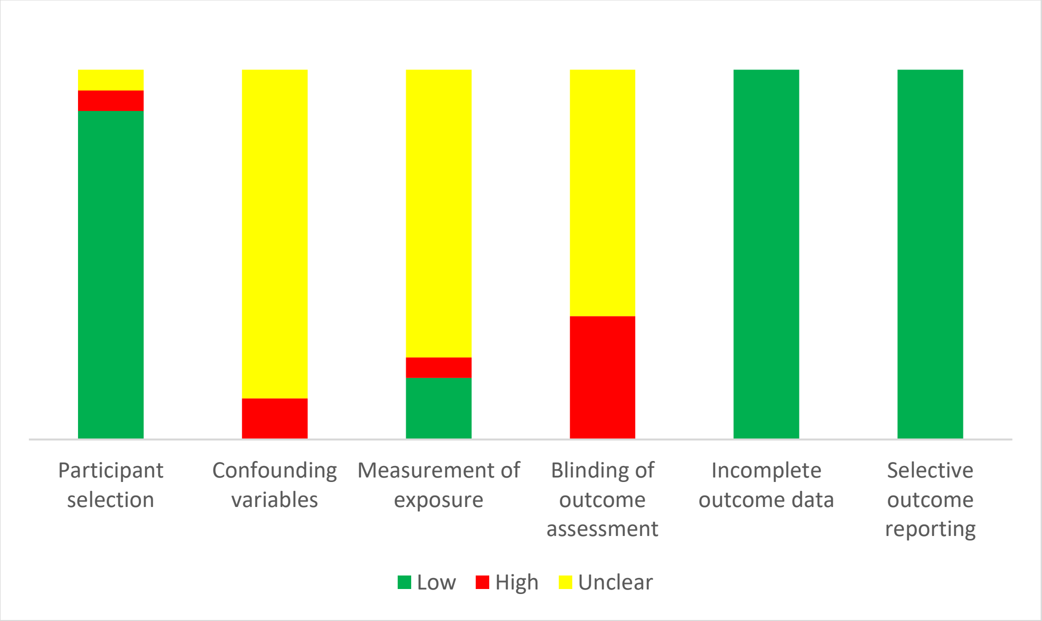

*Using Risk of Bias Assessment Tool for Non-randomized Studies (RoBANS)[10]

B. Risk of bias assessment* by study

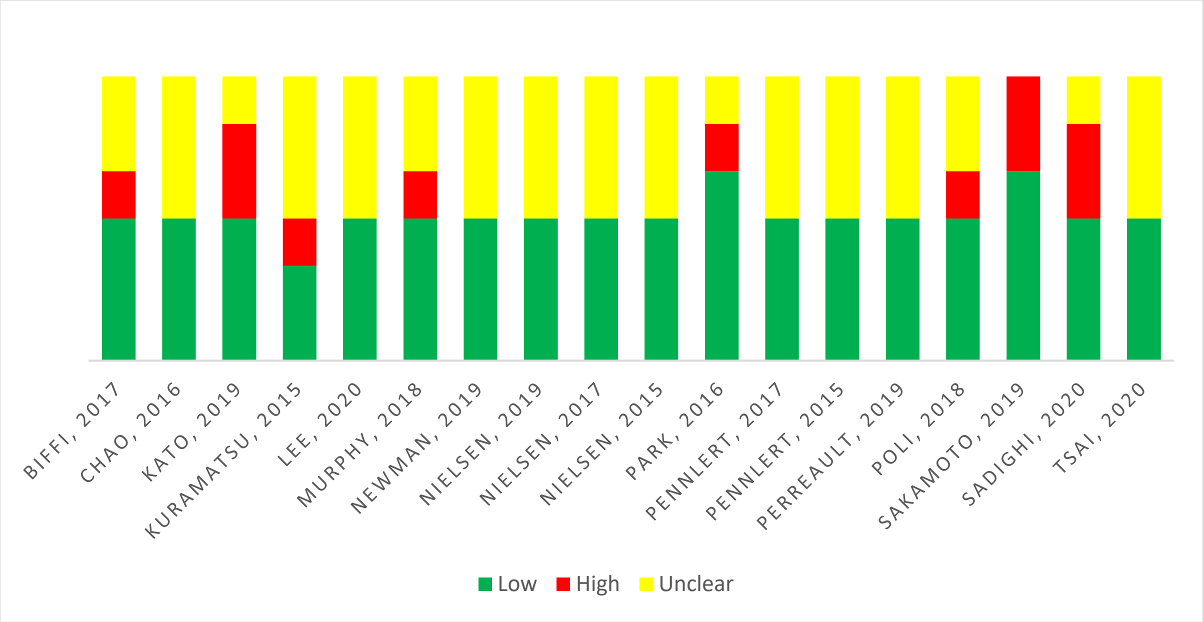

*Using Cochrane Collaboration's tool for assessing risk of bias[11]

Supplementary Figure S2: Risk of bias assessment for included randomised controlled trials

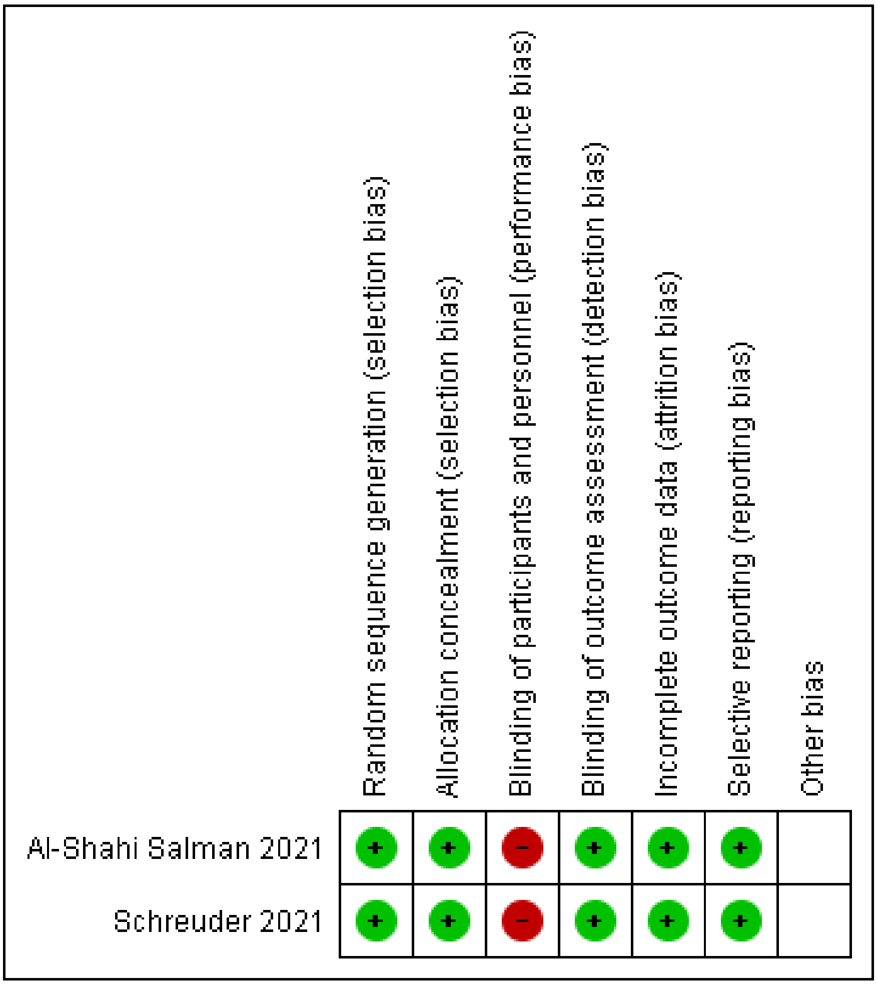

MOOSE Checklist for Meta-analyses of Observational Studies

Item No	Recommendation	Reported on Page No
Reporting of background should include		
1	Problem definition	4
2	Hypothesis statement	N/A
3	Description of study outcome(s)	6
4	Type of exposure or intervention used	6
5	Type of study designs used	8-9
6	Study population	5, 8-9
Reporting of search strategy should include		
7	Qualifications of searchers (eg, librarians and investigators)	6
8	Search strategy, including time period included in the synthesis and key words	6-7
9	Effort to include all available studies, including contact with authors	7
10	Databases and registries searched	6
11	Search software used, name and version, including special features used (eg, explosion)	N/A
12	Use of hand searching (eg, reference lists of obtained articles)	8
13	List of citations located and those excluded, including justification	Figure 1
14	Method of addressing articles published in languages other than English	N/A
15	Method of handling abstracts and unpublished studies	6-7
16	Description of any contact with authors	7
Reporting of methods should include		
17	Description of relevance or appropriateness of studies assembled for assessing the hypothesis to be tested	8<br>Figure 1
18	Rationale for the selection and coding of data (eg, sound clinical principles or convenience)	6, 8
19	Documentation of how data were classified and coded (eg, multiple raters, blinding and interrater reliability)	6-7
20	Assessment of confounding (eg, comparability of cases and controls in studies where appropriate)	7
21	Assessment of study quality, including blinding of quality assessors, stratification or regression on possible predictors of study results	7, 14
22	Assessment of heterogeneity	7
23	Description of statistical methods (eg, complete description of fixed or random effects models, justification of whether the chosen models account for predictors of study results, dose-response models, or cumulative meta-analysis) in sufficient detail to be replicated	7
24	Provision of appropriate tables and graphics	Tables 1, Figs 1-4
Reporting of results should include		
25	Graphic summarizing individual study estimates and overall estimate	Figs 2-4

26	Table giving descriptive information for each study included	Supp. Table S2
27	Results of sensitivity testing (eg, subgroup analysis)	13, Supp. Table S4
28	Indication of statistical uncertainty of findings	9-13, Figs 2-4

PRISMAL 2020 Checklist

Section and Topic	Item #	Checklist item	Location where item is reported
**TITLE**			
Title	1	Identify the report as a systematic review.	Title page
**ABSTRACT**			
Abstract	2	See the PRISMA 2020 for Abstracts checklist.	2-3
**INTRODUCTION**			
Rationale	3	Describe the rationale for the review in the context of existing knowledge.	5
Objectives	4	Provide an explicit statement of the objective(s) or question(s) the review addresses.	5
**METHODS**			
Eligibility criteria	5	Specify the inclusion and exclusion criteria for the review and how studies were grouped for the syntheses.	5-6, 7
Information sources	6	Specify all databases, registers, websites, organisations, reference lists and other sources searched or consulted to identify studies. Specify the date when each source was last searched or consulted.	6
Search strategy	7	Present the full search strategies for all databases, registers and websites, including any filters and limits used.	Supp. Table S2
Selection process	8	Specify the methods used to decide whether a study met the inclusion criteria of the review, including how many reviewers screened each record and each report retrieved, whether they worked independently, and if applicable, details of automation tools used in the process.	6
Data collection process	9	Specify the methods used to collect data from reports, including how many reviewers collected data from each report, whether they worked independently, any processes for obtaining or confirming data from study investigators, and if applicable, details of automation tools used in the process.	6-7
Data items	10a	List and define all outcomes for which data were sought. Specify whether all results that were compatible with each outcome domain in each study were sought (e.g. for all measures, time points, analyses), and if not, the methods used to decide which results to collect.	7, Table 1
	10b	List and define all other variables for which data were sought (e.g. participant and intervention characteristics, funding sources). Describe any assumptions made about any missing or unclear information.	7, Supp. Table S3
Study risk of bias assessment	11	Specify the methods used to assess risk of bias in the included studies, including details of the tool(s) used, how many reviewers assessed each study and whether they worked independently, and if applicable, details of automation tools used in the process.	7, 14
Effect measures	12	Specify for each outcome the effect measure(s) (e.g. risk ratio, mean difference) used in the synthesis or presentation of results.	7
Synthesis methods	13a	Describe the processes used to decide which studies were eligible for each synthesis (e.g. tabulating the study intervention characteristics and comparing against the planned groups for each synthesis (item #5)).	Fig 1
	13b	Describe any methods required to prepare the data for presentation or synthesis, such as handling of missing summary statistics, or data conversions.	7
	13c	Describe any methods used to tabulate or visually display results of individual studies and syntheses.	Table 1;

Section and Topic	Item #	Checklist item	Location where item is reported
			Figs 2-4
	13d	Describe any methods used to synthesize results and provide a rationale for the choice(s). If meta-analysis was performed, describe the model(s), method(s) to identify the presence and extent of statistical heterogeneity, and software package(s) used.	7
	13e	Describe any methods used to explore possible causes of heterogeneity among study results (e.g. subgroup analysis, meta-regression).	7, 13, Supp. Table S4
	13f	Describe any sensitivity analyses conducted to assess robustness of the synthesized results.	13, Supp. Table S4
Reporting bias assessment	14	Describe any methods used to assess risk of bias due to missing results in a synthesis (arising from reporting biases).	7, 13
Certainty assessment	15	Describe any methods used to assess certainty (or confidence) in the body of evidence for an outcome.	7
**RESULTS**			
Study selection	16a	Describe the results of the search and selection process, from the number of records identified in the search to the number of studies included in the review, ideally using a flow diagram.	8, Fig 1
	16b	Cite studies that might appear to meet the inclusion criteria, but which were excluded, and explain why they were excluded.	8, Fig 1
Study characteristics	17	Cite each included study and present its characteristics.	8-9, Suppl. Table S3
Risk of bias in studies	18	Present assessments of risk of bias for each included study.	13, Supp. Fig S1
Results of individual studies	19	For all outcomes, present, for each study: (a) summary statistics for each group (where appropriate) and (b) an effect estimate and its precision (e.g. confidence/credible interval), ideally using structured tables or plots.	Figs 2-4
Results of syntheses	20a	For each synthesis, briefly summarise the characteristics and risk of bias among contributing studies.	9-13
	20b	Present results of all statistical syntheses conducted. If meta-analysis was done, present for each the summary estimate and its precision (e.g. confidence/credible interval) and measures of statistical heterogeneity. If comparing groups, describe the direction of the effect.	9-13, Figs 2-4
	20c	Present results of all investigations of possible causes of heterogeneity among study results.	9-13, Figs 2-4
	20d	Present results of all sensitivity analyses conducted to assess the robustness of the synthesized results.	13, Supp. Table S4
Reporting biases	21	Present assessments of risk of bias due to missing results (arising from reporting biases) for each synthesis assessed.	-
Certainty of evidence	22	Present assessments of certainty (or confidence) in the body of evidence for each outcome assessed.	9-13
**DISCUSSION**			

Section and Topic	Item #	Checklist item	Location where item is reported
Discussion	23a	Provide a general interpretation of the results in the context of other evidence.	14-16
	23b	Discuss any limitations of the evidence included in the review.	16-17
	23c	Discuss any limitations of the review processes used.	16-17
	23d	Discuss implications of the results for practice, policy, and future research.	17
**OTHER INFORMATION**			
Registration and protocol	24a	Provide registration information for the review, including register name and registration number, or state that the review was not registered.	5
	24b	Indicate where the review protocol can be accessed, or state that a protocol was not prepared.	5
	24c	Describe and explain any amendments to information provided at registration or in the protocol.	N/A
Support	25	Describe sources of financial or non-financial support for the review, and the role of the funders or sponsors in the review.	18
Competing interests	26	Declare any competing interests of review authors.	18
Availability of data, code and other materials	27	Report which of the following are publicly available and where they can be found: template data collection forms; data extracted from included studies; data used for all analyses; analytic code; any other materials used in the review.	5
